# Enhanced De Novo Lipid Synthesis Mediated by FASN Induces Chemoresistance in Colorectal Cancer

**DOI:** 10.3390/cancers15030562

**Published:** 2023-01-17

**Authors:** Lingyu Han, Weixing Dai, Wenqin Luo, Li Ye, Hongsheng Fang, Shaobo Mo, Qingguo Li, Ye Xu, Renjie Wang, Guoxiang Cai

**Affiliations:** 1 Department of Colorectal Surgery, Fudan University Shanghai Cancer Center, Shanghai 200032, China; 2 Department of Oncology, Shanghai Medical College, Fudan University, Shanghai 200032, China

**Keywords:** colorectal cancer, oxaliplatin, fatty acid metabolism, drug resistance, FASN

## Abstract

**Simple Summary:**

Resistance to oxaliplatin threatens the prognosis in of colorectal cancer (CRC). As previous studies have aroused interest in fatty acid metabolism in cancer, we determined whether fatty acid biosynthesis contribute to oxaliplatin resistance in CRC. By leveraging the GEO databases, FASN gene signatures was correlated with the response to oxaliplatin-based chemotherapy and poor prognosis. Additionally, FASN expression was positively related with oxaliplatin resistance in vitro. Then, Orlistat, a typical FASN inhibitor, was applied to attenuate the resistance to oxaliplatin in cell culture and xenograft models. Additionally, the combination of the FASN inhibitor and oxaliplatin significantly increased cell cycle arrest and facilitated apoptosis, partly due to the diminished phosphorylation of the MAPK/ERK and PI3K/AKT pathways. Our study revealed that FASN enhanced resistance to oxaliplatin in CRC. Inhibition of FASN could rescue the response to oxaliplatin by regulating MAPK/ERK and PI3K/AKT pathways.

**Abstract:**

Background: Oxaliplatin is one of the most widely used chemotherapy drugs for colorectal cancer (CRC). Resistance to oxaliplatin threatens the prognosis of CRC. Since previous studies have aroused interest in fatty acid metabolism in cancer, in this study, we determined whether fatty acid biosynthesis and the related regulating mechanism contribute to oxaliplatin resistance in CRC. Methods: The effect of the fatty acid synthase (FASN) and its inhibitor Orlistat was characterized in Gene Expression Omnibus (GEO) databases, oxaliplatin-resistant cell lines, and xenografts. MRNA-seq and analysis identified related pathway changes after the application of Orlistat, which was verified by Western blotting. Results: By leveraging the GEO databases, FASN and closely related gene signatures were identified as being correlated with the response to oxaliplatin-based chemotherapy and poor prognosis. Additionally, FASN-upregulated expression promoted oxaliplatin resistance in CRC cell lines. We then applied Orlistat, a typical FASN inhibitor, in cell culture and xenograft models of oxaliplatin-resistant CRC, which attenuated the resistance to oxaliplatin. Additionally, the combination of the FASN inhibitor and oxaliplatin significantly increased cell cycle arrest and facilitated apoptosis, partly due to the diminished phosphorylation of the MAPK/ERK and PI3K/AKT pathways. In vivo studies showed that inhibiting fatty acid biosynthesis with Orlistat restrained the growth of xenograft tumors and increased the responsiveness to oxaliplatin. Conclusions: Our study revealed that FASN enhanced resistance to oxaliplatin in CRC. The inhibition of FASN could rescue the response to oxaliplatin by regulating MAPK/ERK and PI3K/AKT pathways.

## 1. Introduction

Colorectal cancer (CRC) ranks in the top three for both new cases and cancer-related deaths [1]. Although the cure rate of early stage CRC has reached nearly 90%, recurrence and metastasis remain significant causes of death in patients with advanced colorectal cancer. Targeted therapy and chemotherapy are frequent and efficient treatments for CRC that improve disease-associated symptoms and long-term survival [2]. On the other hand, due to drug resistance, the efficacy of conventional chemotherapy is limited [3]. These deficiencies limit the effect of drug therapy. Therefore, a new therapeutic scheme is of paramount importance for improving prognosis.

As one of the most popular treatments used in CRC, oxaliplatin is a classic platinum drug that inhibits DNA replication and transcription by promoting the formation of intra- and interstrand DNA breaks [4]. With increasing studies concentrating on metabolic alterations during cancer therapy [5,6,7], a comprehensive therapeutic strategy combining metabolic pathways and chemotherapy may further prevent drug resistance and increase chemosensitivity to oxaliplatin [8,9,10]. In addition to the Warburg effect, fatty acids are an important energy source for tumor cells. According to previous reports, changes in fatty acid metabolism pathways in tumor cells are closely related to the occurrence of chemotherapy resistance [11]. To better adapt to the vigorous energy metabolism caused by rapid growth, fatty acid synthesis in tumor cells becomes more active [12]. However, the mechanism of the action of specific metabolic enzymes in the process of tumor resistance and their corresponding therapeutic targets are still not fully understood in CRC.

Compared with nontransformed cells, cancer cells are more dependent on de novo fatty acid biosynthesis. The expression of fatty acid synthase (FASN) is elevated in multiple cancers, including colorectal cancer [12,13,14]. Furthermore, the potential of FASN to induce tumor invasion and metastasis has also been widely reported [15]. Tadros et al. reported a critical role of fatty acid synthase inhibitors in antagonizing gemcitabine resistance in pancreatic cancer [11].

In this study, we attempted to find a link between abnormal fatty acid synthesis and oxaliplatin resistance in CRC. Our study showed that the pharmacological inhibition of fatty acid synthesis in vivo inhibits and reverses oxaliplatin resistance in colorectal cancer cells by regulating MAPK/ERK and PI3K/AKT pathways.

## 2. Materials and Methods

### 2.1. Public Dataset and Pathway Analysis

GSE195860, GSE28702, and GSE69657, three public microarray datasets of CRC, were downloaded from the Gene Expression Omnibus (GEO) database (https://www.ncbi.nlm.nih.gov/geo/, accessed on 14 September 2022). The data-processing procedure was described in our previous work [16].

### 2.2. Cell Culture and Reagents

The human colorectal cancer cell lines SW480, HCT116, HCT8, HCT15, DLD1, and RKO were purchased from the National Cancer Institute (Bethesda, MD, USA). HCT8 and HCT116 cells were seeded in a 6 cm culture flask with LOHP solution in the medium. The concentration of LOHP was raised from 2 μmol/L to 30 μmol/L by 1 μmol/L every three subcultures. Ultimately, Oxaliplatin-resistant cell lines (HCT8-LOHP and HCT116-LOHP) were cultured in 5 μmol/L LOHP. The two cell lines were utilized for in vitro experiments after culturing in medium without LOHP for 2 weeks. All these cells were cultured in high-glucose Dulbecco’s modified Eagle medium (DMEM; 4.5 mg/L glucose, 4 mM glutamine; Gibco), supplemented with 10% fetal bovine serum (FBS; 10099141, Thermo Fisher Scientific, Waltham, MA, USA), and cultivated in an incubator in a 5% CO_2_ atmosphere at 37 °C.

### 2.3. Gene Silencing and Overexpression with Lentivirus

Plasmids containing an shRNA against FASN and lentiviral vectors for overexpressing FASN were both designed and synthesized by Jiekesaisi (Nanjing, China). A lentiviral packaging kit (Yeasen, Shanghai, China) comprised lentivirus media for plasmid cotransfection into HEK293T cells. After 48 h, CRC cell lines were seeded in 6-well plates and treated with harvested lentivirus. After another 48 h, 5 μg/mL puromycin was added to the cell medium to complete chemical selection.

### 2.4. Application of Fatty Acid Inhibitors and Palmitate

For the application of the FASN synthetase inhibitor Orlistat (O4139-25MG, Merck, Darmstadt, Germany) in vitro, the inhibitor was prepared by referencing the procedure of Browne et al. [17], and 200 µmol/L Orlistat was adopted to treat CRC cells for 48 h when necessary. Another FASN inhibitor, C75 (25–200 µmol/L) (HY-12364, MedChemExpress, Monmouth Junction, NJ, USA), was used to treat CRC cells for 48 h as per a report from Chen et al. [11]. Based on a previous study [18], 800 µmol/L of palmitoleic acid (HY-N1966, MedChemExpress) was applied for 72 h for in vitro experiments.

### 2.5. Quantitative Real-Time PCR (qRT–PCR) and Western Blot Analyses

Total RNA from clinical specimens and CRC cells was isolated using TRIzol Reagent (15596026, Invitrogen, NY, USA). Reverse transcription was performed using PrimeScript RT reagent (RR036A, TaKaRa, Kusatsu, Japan). The mRNA levels of relative genes were assessed by real-time quantitative polymerase chain reaction (RT–qPCR) using a cDNA QuantStudio (TM) 7 Flex System, with β-actin as a control. Relative primer sequence is listed in Supplementary Table S1.

Western blotting was performed using cell lysates. The primary antibodies used were as follows: anti-β-actin (4970, Cell Signaling, Danvers, MA, USA), anti-FASN (10624-2-AP, Proteintech), anti-AKT serine/threonine kinase (AKT) (10176-2-AP, Proteintech, Rosemont, IL, USA), anti-p-AKT (66444-1-Ig, Proteintech), anti-p38 (14064-1-AP, Proteintech), anti-p-p38 (28796-1-AP, Proteintech), anti-extracellular signal-regulated kinase (ERK) (16443-1-AP, Proteintech), anti-p-ERK (28733-1-AP, Proteintech), and anti-p-PI3K (4228, Cell Signaling Technology). The relevant quantification of Western blot was completed using Image J (1.53v).

### 2.6. The mRNA-Seq and Analysis

Oxaliplatin-resistant cells were cultured for 72 h with the corresponding medium in a 10 cm plate and collected using TRIzol reagent (15596026, Invitrogen, Carlsbad, CA, USA). All the samples were sent to Shanghai Genechem Co., Ltd. (Shanghai, China) for data analysis. Three independent repetitive samples were validated. After qualification testing, RNA sequencing libraries were established, which were used to analyze the massive ends of cDNA. In brief, biotinylated poly (dT) primers (5′d PO4 [(T)12-18] 3′) were applied to purify the polyadenylated RNA from the total RNA isolated by TRIzol reagent. Afterward, it was reverse-transcribed to cDNA, then fragmented to a size ranging from 150 to 600 bp. Streptavidin magnetic beads were adopted to capture the biotinylated ends, which were subsequently added to the modified adapters. Polymerase chain reaction (annealing: 55–65 °C, 5 min, 4 °C, 2 min; polymerization: 10–50 °C, 10–90 min; deactivation: 85 °C, 10 min) was conducted to amplify the aforementioned sequencing libraries, purification was performed using SPRI beads, and then sequencing was performed (HiSeq2000, Illumina, San Diego, CA, USA). After normalization, genes with a false discovery rate (FDR) of <0.05 and |log2 fc| > 1.5 were deemed differentially expressed.

### 2.7. Cell-Viability, Colony-Formation, and Migration Assays

Cell viability was determined using MTT assay. Briefly, 1*103 colorectal cells were seeded into 96-well plates per well with 5 repetitions of each group. After 12 h of cell attachment, 10 µL Cell Counting Kit-8 (CK04, DOJINDO, Kumamoto, Japan) reagent (Cell Counting Kit, Yeasen) was added to 100 µL cell culture medium without FBS per well. Then, the plate was placed in an incubator at 37 °C for 2 h in the dark. Finally, the absorbance value of the whole plate was measured at 450 nm using a Biotek system. The curves of cell proliferation were plotted each day. As for colony-formation assays, 500 CRC cells were seeded into 6-well plates, and the colonies formed were counted 1 week later.

Transwell assays were performed by seeding 4*104–8*104 cells into the upper chamber (CLS3464, Corning Costar, Corning, NY, USA) with no FBS supplementation, while 500 µL DMEM with 10% FBS was added to the lower chamber. After 72 h of culture, migrated cells were fixed with 4% paraformaldehyde (G1101, Servicebio, Wuhan, Hubei, China), stained with crystal violet staining solution (C0121, Beyotime, Shanghai, China), and counted under a microscope.

### 2.8. Quantification of Triacylglycerol and Neutral Lipids

For the quantitative estimation of triglycerides in cells, a Triglyceride Assay Kit (ab65336, Abcam, Cambridge, UK) was used in accordance with the manufacturer’s protocols. The lipophilic fluorescence dye BODIPY 493/503 (D3922, Invitrogen) was applied to stain the neutral lipid droplets, and flow cytometry (MoFlo XDP, Beckman Coulter, Pasadena, CA, USA) was conducted to quantify the neutral lipid content.

### 2.9. Cell-Cycle, ROS, and Apoptosis Assays

Following the manufacturer’s protocol, the cell cycle (Cell Cycle Staining Kit CCS012, Multi Sciences), ROS (the Reactive Oxygen Species Assay Kit, Beyotime), and apoptosis (Annexin V-PE/7-AAD Apoptosis Detection Kit, Yeasen) were calculated. Briefly, after staining with 7-AAD viability staining solution and rh Annexin V/PE simultaneously, apoptotic cells were examined by flow cytometry, and a two-color dot plot was drawn. Data analysis was carried out by BD AccuriR C6 (BD Biosciences).

Caspase-3/7 activity was detected by the Caspase 3/7 Activity Apoptosis Assay Kit *Green Fluorescence (Sangon Biotech). According to the manufacturer’s protocol, the fluorescence intensity of Rh110 generated by Caspase 3/7 was measured at Ex/Em = 490/525.

### 2.10. In Vivo Studies

All animal studies were approved by the Ethical Committee of Fudan University Shanghai Cancer Center. Four-week-old Balb/c nude mice were purchased and housed in flow cabinets under specific pathogen-free conditions. After 4 days of adaptation, the mice were randomly assigned to each group. CRC cells (1.2*106 HCT116-LOHP cells) suspended in PBS were injected subcutaneously into both sides of the flanks of each mouse to establish the CRC xenograft model. For Orlistat and oxaliplatin treatment, mice were divided into four groups when the tumor volume was close to 40 mm3. For fatty acid inhibition, treatment was prepared, as described by Kridel et al. [19], and Orlistat was intraperitoneally injected into nude mice at a concentration of 240 mg/kg. A total of 10 milligrams per kilogram of oxaliplatin 2 times a week, 240 mg/kg of Orlistat 5 times a week, or the corresponding solvent was given by intraperitoneal injection. After harvesting, tumor volume was calculated according to the following formula: (longest diameter × shortest diameter^2^)/2.

### 2.11. Statistical Analysis

R software (R version 3.2.5, https://www.r-project.org/) was used for statistical analysis. Data are depicted as the mean ± standard deviation. The Wilcoxon rank-sum test or Student’s t-test was applied to compare the differences between two groups. Overall survival (OS) was depicted by the Kaplan–Meier method, and the significant differences were compared with the log-rank test. OS was defined as the time from treatment initiation to death or last follow-up. The relationship between FASN expression and other genes was evaluated by the Spearman rank correlation test. *p* < 0.05 was deemed statistically significant.

## 3. Results

### 3.1. FASN Expression Was Associated with Worse Prognosis and Poor Oxaliplatin Therapy Response in Colorectal Cancer Patients

**Figure 1:**
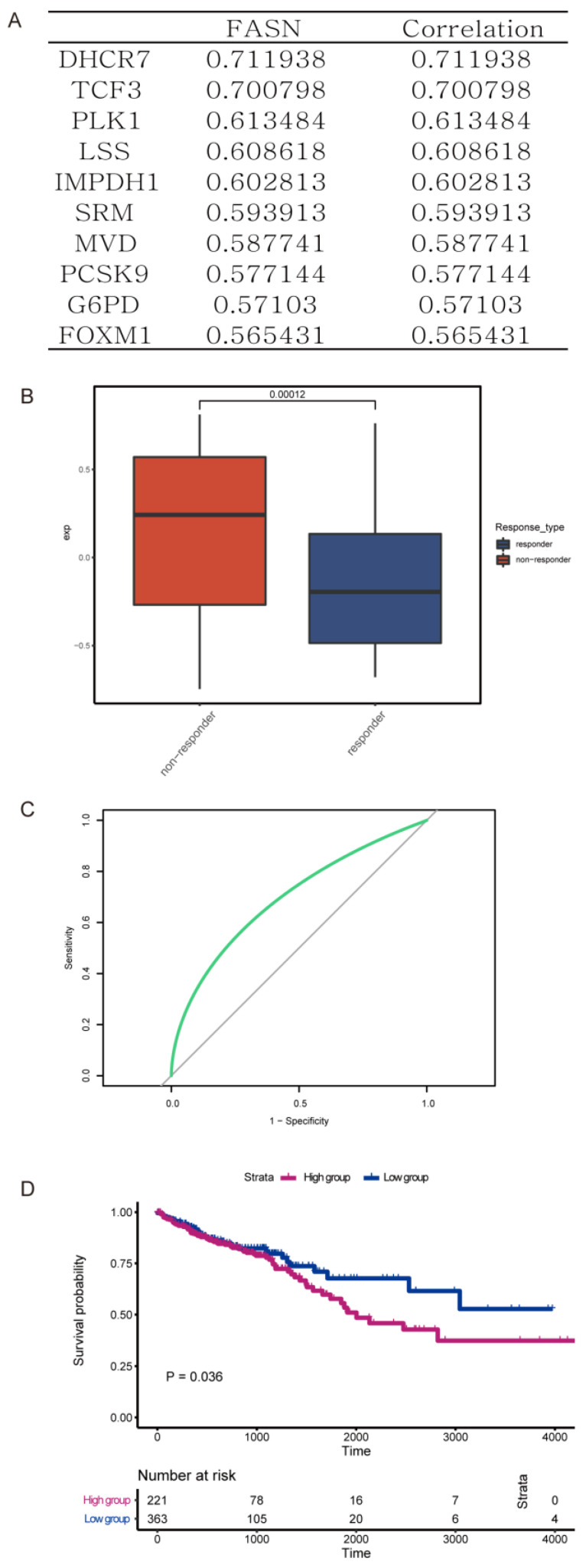
FASN biosynthesis correlates with progression and resistance to chemotherapy in CRC. (**A**) 10 crucial genes closely positively correlated with expression of FASN. (**B**) Compared with the response to oxaliplatin, expression of the FASN gene signature in those resistant to oxaliplatin was higher. (**C**,**D**) The ROC curve (**C**) and Kaplan–Meier overall survival curves (**D**) of patients with different levels of FASN gene signature (*p* = 0.036, AUC = 0.696).

FASN, one of the key enzymes in the de novo fatty acid biosynthesis, catalyzes the synthesis of palmitate by acetyl-CoA and malonyl-CoA. To explore the mechanistic function of fatty acid biosynthesis, especially that of FASN, in colorectal cancer progression, we analyzed the expression of multiple genes from the GEO database (GSE195860, GSE28702, and GSE69657). Samples were divided into two quartiles with the highest and lowest expression, and relative gene expression was compared. Based on the results, there were 10 crucial genes closely correlated with the expression of FASN (Figure 1A). Therefore, we identified these genes and FASN as an FASN gene signature. Compared with those who responded to chemotherapy with oxaliplatin, those CRCs that were resistant to oxaliplatin had a higher expression of the FASN gene signature (Figure 1B). Additionally, we observed that this FASN signature could also be utilized to predict patient prognosis (*p* = 0.036), and the relative AUC was 0.696 (Figure 1C,D). Collectively, these data demonstrated that FASN was upregulated in a patient with oxaliplatin resistance and a worse prognosis.

### 3.2. De Novo Fatty Acid Biosynthesis Rescued Colorectal Cancer Cells from Oxaliplatin

As a key enzyme of the fatty acid biosynthetic pathway, the expression of FASN was related to total intracellular fatty acids. After the knockdown of FASN, MTT assays confirmed that the proliferation of oxaliplatin-resistant HCT116 cell lines (HCT116-LOHP) and HCT8 cell lines (HCT8-LOHP) were suppressed (Supplementary Figure S1A,B, Relative intensity ratio of each band on western blot see Table S4). Transwell assays demonstrated that the migration of HCT8 and HCT116 cells was inhibited (Supplementary Figure S1C). Additionally, FASN overexpression lentivirus was transfected into CRC cells to establish stable cell lines. The overexpression of FASN promoted the proliferation and migration of HCT116 and HCT8 cells (Supplementary Figure S1D–F, Relative intensity ratio of each band on western blot see Table S5).

**Figure 2:**
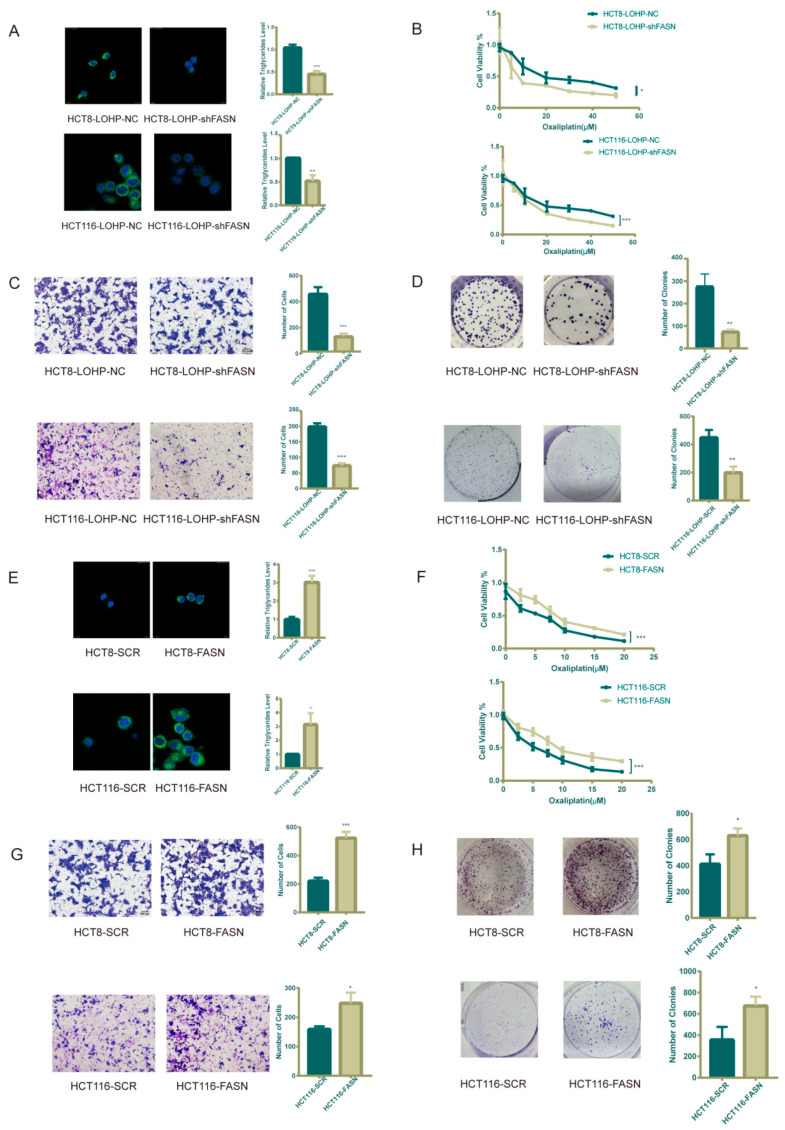
De novo lipid biosynthesis makes CRC cell lines more resistant to oxaliplatin. (**A**) Intracellular neutral lipid droplets and triglycerides were decreased in oxaliplatin-resistant cells after FASN knockdown. The storage of lipids is shown as bodipy 493/503 staining of neutral lipid droplets. (**B**) Upon treatment with same dose of oxaliplatin, there was a significant fold decrease in proliferation in the FASN knockdown CRC cells. (**C**) Transwell assay indicated that FASN knockdown inhibited migration of oxaliplatin-resistant CRC cells previously treated with 20 μM oxaliplatin for 72 h. (**D**) FASN knockdown and relative wild-type oxaliplatin-resistant CRC cells were previously treated with 20 μM oxaliplatin for 72 h. Clonogenic assay was performed as 500 cells seeded in 6-well plates for 7 days and counting. (**E**,**F**) Intracellular neutral lipid droplets, triglycerides, and IC50 of oxaliplatin increased after FASN overexpression. (**G**) Transwell assay indicated that FASN overexpression improved migration of CRC cells previously treated with 5 μM oxaliplatin for 72 h. (**H**) There was a distinct increase in colony formation in FASN-overexpression CRC cells. * *p* ≤ 0.05, ** *p* ≤ 0.01, and *** *p* ≤ 0.001.

To better understand the mechanism by which FASN regulates oxaliplatin resistance in CRC cell lines, we explored the effect of FASN on lipid metabolism and the IC50 of oxaliplatin. Accordingly, we assumed that FASN impairs the sensitivity of cells to oxaliplatin through the accumulation of intracellular lipid droplets. We demonstrated that knocking down FASN expression in HCT116-LOHP and HCT8-LOHP cells using lentiviral vectors inhibited the accumulation of triglycerides and lipid droplets (Figure 2A) and lowered the IC50 of oxaliplatin in the CRC cell lines (HCT8-LOHP-NC 22.34 μmol/L vs. HCT8-LOHP-shFASN 8.69 μmol/L, *p* = 0.024; HCT116-LOHP-NC 22.24 μmol/L vs. HCT116-LOHP-shFASN 13.7 μmol/L, *p* = 0.013) (Figure 2B). Additionally, knocking down FASN significantly suppressed the migration and proliferation of oxaliplatin-resistant cell lines after treatment with oxaliplatin (Figure 2C,D). On the other hand, FASN overexpression in both HCT116 and HCT8 cell lines promoted the accumulation of triglycerides and lipid droplets (Figure 2E) and elevated the IC50 of oxaliplatin (HCT8-SCR 4.66 μmol/L vs. HCT8-FASN 7.79 μmol/L, *p* = 0.004; HCT116-SCR 3.49 μmol/L vs. HCT116-FASN 7.60μmol/L, *p* = 0.045) (Figure 2F). Increasing the expression of FASN in these two CRC cell lines also promoted the ability to invade and form clones against oxaliplatin (Figure 2G,H).

### 3.3. Pharmacological Inhibition of FASN Synergistically Enhanced the Antiproliferative Effect of Oxaliplatin in Human Colorectal Cancer Cells

**Figure 3:**
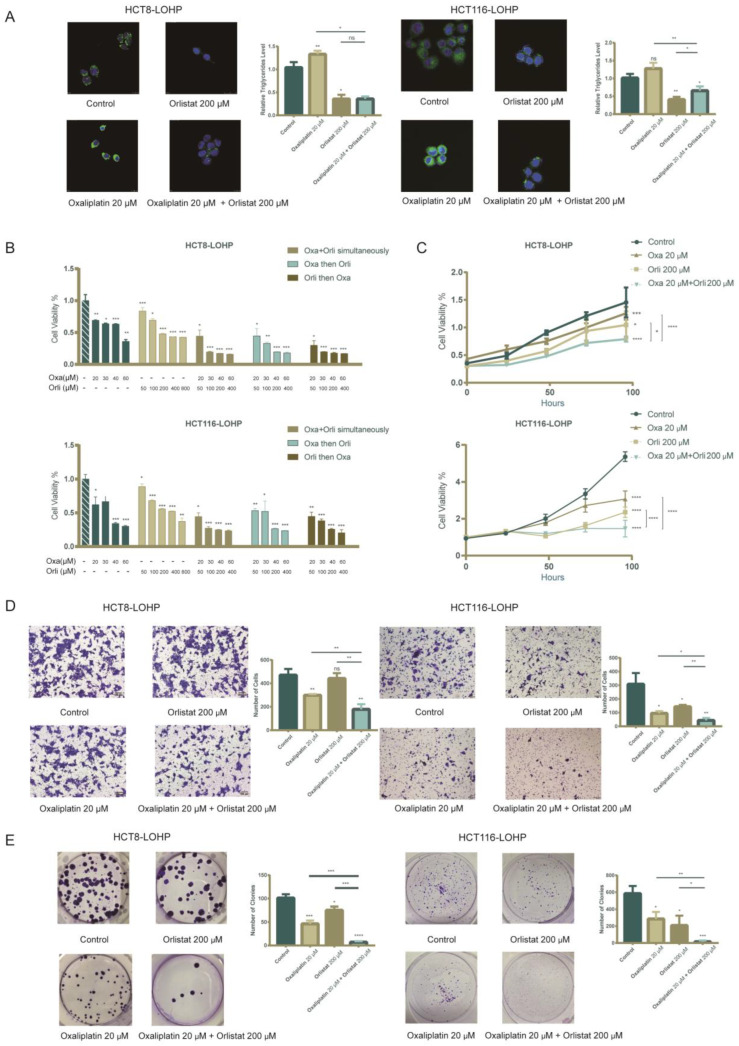
Synergistic inhibition fatty acid biosynthesis combined with oxaliplatin diminished cell viability, clonogenicity, and migration. (**A**) There was a significant decrease in intracellular neutral lipid droplets and triglycerides after treatment with Orlistat for 72 h. (**B**) Cell viability in HCT116-LOHP and HCT8-LOHP cells upon different treatment schemes: oxaliplatin, Orlistat, combination simultaneously, sequentially, and reverse-sequentially for 72 h. MTT assay was performed to assess cell viability. (**C**) There was a significant decrease in cell proliferation after treatment with Orlistat, and combination of Orlistat and oxaliplatin robustly enhanced the anti-proliferation ability of oxaliplatin. (**D**) Transwell assay depicted that sequential treatment with combination of Orlistat and oxaliplatin for 72 h effectively inhibited migration of CRC cell lines. (**E**) Clonogenic assay indicated that additional treatment of Orlistat promoted inhibitory effect of oxaliplatin on cell proliferation. * *p* ≤ 0.05, ** *p* ≤ 0.01, *** *p* ≤ 0.001, **** *p* ≤ 0.0001, ns: no significance.

Next, we evaluated whether systematic pharmacological targeting of FASN would promote the sensitivity of CRC cell lines to oxaliplatin. Orlistat, an inhibitor of the thioesterase domain of FASN, was administered to colorectal cancer cell lines. We demonstrated that Orlistat could inhibit proliferation and migration in several CRC cell lines (Supplementary Figure S2). In HCT116-LOHP and HCT8-LOHP cells, Orlistat significantly reduced the cellular neutral lipid droplets and triglyceride accumulation in treated cells compared with control cells (Figure 3A). In addition, there was an antiproliferative effect in all cell lines in a concentration-dependent manner. The MTT analysis of CRC cell lines exposed to simultaneous/sequential doses of oxaliplatin and Orlistat exhibited a synergistic reduction in cell survival regardless of whether the two drugs were used simultaneously or sequentially (Figure 3B). Additionally, the growth rates of both HCT116-LOHP and HCT8-LOHP cells decreased under treatments with oxaliplatin and Orlistat (*p* < 0.05) (Figure 3C). After 72 h of treatment, migration inhibition in cells treated with additional Orlistat was more intense than in cells treated with oxaliplatin alone (Figure 3D). Furthermore, to determine whether oxaliplatin and Orlistat inhibited tumor growth in the long term, we performed clonogenic assays, and sequential pharmacological treatment demonstrated a robust decrease in the formation of colonies (Figure 3E).

Additionally, we explored whether exogenous fatty acids could rescue colorectal cancer cells from oxaliplatin. To achieve this, we simultaneously treated colorectal cancer cell lines with Orlistat and palmitate. Palmitate could partly rescue CRC cell lines from the inhibition of growth due to Orlistat. Then, we further divided these cell lines into oxaliplatin-treated and control groups (Supplementary Figure S3). As we previously hypothesized, lipid accumulation in CRC cells partly adjusted the resistance of CRC cells to chemotherapy.

In addition to Orlistat, we also applied C75, another inhibitor of FASN [11], to oxaliplatin-resistant cells. Similar to Orlistat, C75 sequentially combined with oxaliplatin inhibited oxaliplatin-resistant cell growth (Supplementary Figure S4).

### 3.4. The Combination of Orlistat and Oxaliplatin Caused Cell-Cycle Arrest and Facilitated Oxaliplatin-Induced Apoptosis

**Figure 4:**
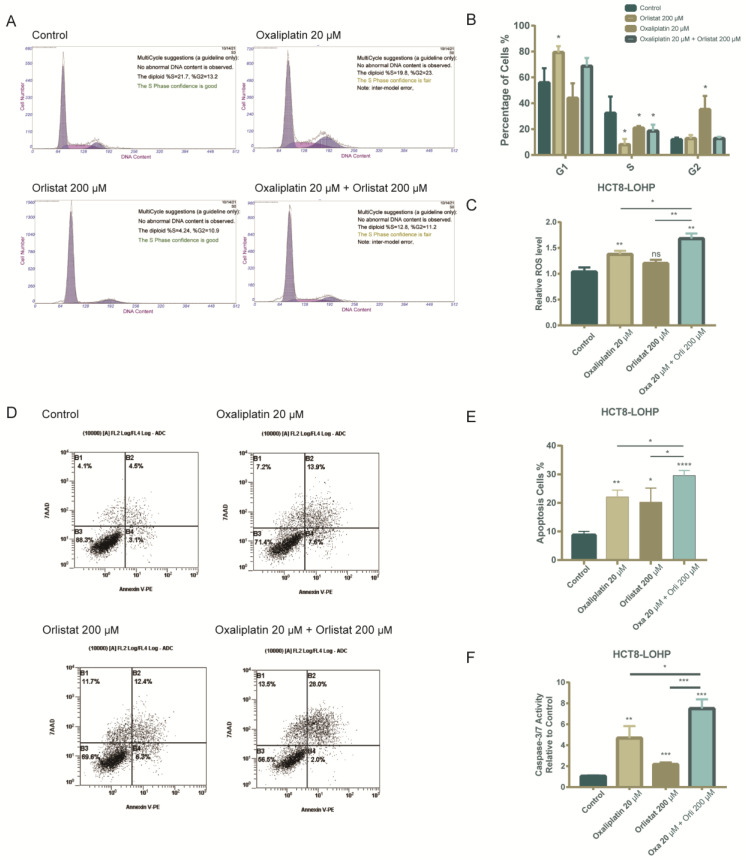
Oxaliplatin combined with Orlistat induced cell-cycle arrest and increased ROS levels and apoptosis in HCT8-LOHP cell lines. (**A**,**B**) Cell-cycle analysis by flow cytometry indicated that treatment with oxaliplatin, Orlistat, and drug combination led to G1 arrest. Additionally, the change in each phase of cell cycle in each group was analyzed by one-way ANOVA with the Dunnett post hoc test. (**C**) Reactive oxygen species (ROS) was evaluated using the Reactive Oxygen Species Assay Kit (Beyotime). The result indicated that combination of Orlistat and oxaliplatin significantly increased ROS levels in HCT8-LOHP cell lines. (**D**,**E**) Apoptosis level, indicated by staining cells with Annexin V/7-AAD in each group in indicated treatments, was compared with control by one-way ANOVA with Dunnett’s post hoc test, respectively. (**F**) Cellular caspase-3/7 activity was determined by the Caspase 3/7 Activity Apoptosis Assay Kit *Green Fluorescence (Sangon Biotech) and expressed relative to the control. Cells were treated for 72 h. Relative caspase activity upon indicated treatments was compared with the control by one-way ANOVA with Tukey’s post hoc test. * *p* ≤ 0.05, ** *p* ≤ 0.01, *** *p* ≤ 0.001, **** *p* ≤ 0.0001, ns: no significance.

We next analyzed whether Orlistat alone and combined with oxaliplatin could influence cell-cycle progression by staining with propidium iodide and subsequent flow cytometry. HCT8-LOHP cells demonstrated G1 arrest after exposure to Orlistat alone or in combination with oxaliplatin (Figure 4A,B). Furthermore, the simultaneous use of Orlistat and oxaliplatin increased the apoptosis of HCT8-LOHP cell lines. As reported previously, increasing ROS levels may help oxaliplatin-resistant colorectal cancer cells to progress and evade the cell-killing effects of oxaliplatin. However, as the ROS level continues to increase, apoptosis is induced [11]. Compared with treatment with oxaliplatin, combination treatment with oxaliplatin and Orlistat induced a further increase in ROS levels (Figure 4C) and more apoptotic cells in colorectal cancer cell lines (Figure 4D,E). Next, we evaluated whether apoptosis is enhanced by Orlistat, oxaliplatin, or their combination. We observed a significantly increased activity of caspase 3/7, a marker of apoptosis, upon the treatment of HCT8-LOHP cells with the combination of oxaliplatin and Orlistat compared to oxaliplatin alone (Figure 4F).

### 3.5. Orlistat Promoted Oxaliplatin-Induced Cytotoxicity by Inhibiting Phosphorylation of the MAPK/ERK and PI3K/AKT Pathways

**Figure 5:**
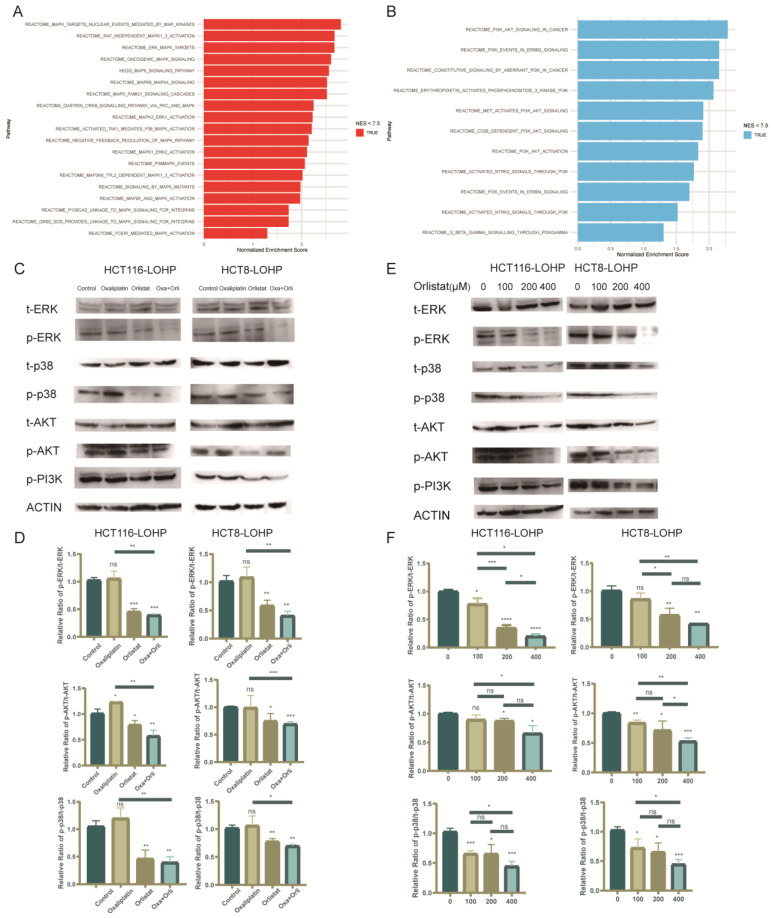
Phosphorylation of MAPK/ERK and PI3K/AKT pathways was involved in the process by which Orlistat enhances anti-proliferation effect of oxaliplatin. (**A**,**B**) Genome-wide RNA sequencing (RNA-seq) analysis was performed on HCT8-LOHP cells. Gene ontology (GO) analysis compared untreated cells with Orlistat treatment schemes and depicted the most significant change in signaling pathways. Compared with cell lines treated with Orlistat, MAPK/ERK (**A**) and PI3K/AKT (**B**) pathways were activated more. (**C**,**D**) There was a distinct change in phosphorylation of MAPK/ERK and PI3K/AKT pathways in cell lines exposed to oxaliplatin, Orlistat, and both. Western blot analysis depicted that whether oxaliplatin was utilized or not, Orlistat could inhibit phosphorylation of MAPK/ERK and PI3K/AKT pathways. Relative intensity ratio of each band on western blot see Table S2. (**E**,**F**) Under 20 µM oxaliplatin treatment, phosphorylation levels in 116-LOHP and HCT8-LOHP cell lines decreased with increasing concentration gradients of Orlistat. Relative intensity ratio of each band on western blot see Table S3. The uncropped blots are shown in Figure S5. * *p* ≤ 0.05, ** *p* ≤ 0.01, *** *p* ≤ 0.001, **** *p* ≤ 0.0001, ns: no significance.

To further investigate the mechanism by which FASN regulates oxaliplatin resistance, we performed genome-wide RNA sequencing (RNA-seq) analysis of HCT8-LOHP cells on the oxaliplatin-treated group and the oxaliplatin combined with Orlistat group. According to gene ontology (GO) analysis, the MAPK/ERK (Figure 5A) and PI3K/AKT (Figure 5B) pathways were two significantly inhibited signaling pathways after the addition of Orlistat. Chen et al. found that the activation of the MAPK and AKT signaling pathways promoted CRC growth and epithelial-to-mesenchymal transition (EMT) [20]. Previous studies demonstrated that inhibition of the MAPK and PI3K/AKT pathways increased the sensitivity to oxaliplatin [21,22]. Therefore, we elucidated the changes in the MAPK/ERK and PI3K/AKT pathways induced by Orlistat (Figure 5C,D). The expression of phosphorylated ERK, p38, AKT, and PI3K in HCT116-LOHP and HCT8-LOHP cells was inhibited by Orlistat in a dose-dependent manner (Figure 5E,F).

### 3.6. Decreased Tumor Burden in a Xenograft-Implantation Model of Colorectal Cancer by the Sequential Combination of Orlistat and Oxaliplatin

**Figure 6:**
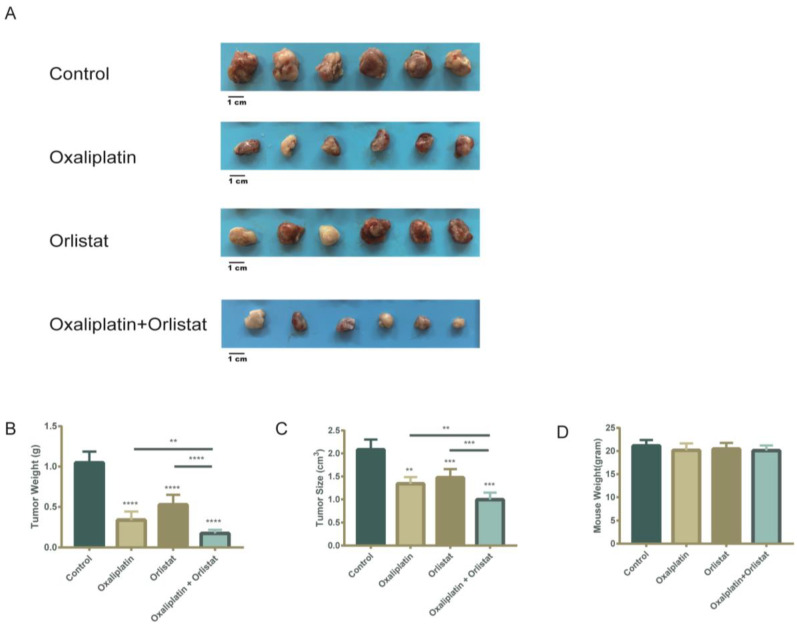
In vivo sequential combination of Orlistat and oxaliplatin restrained tumor growth more. (**A**) Xenograft implantation was collected 30 days after subcutaneous injection of HCT116-LOHP cell lines. A total of 240 mg/kg of Orlistat five times a week, 10 mg/kg of oxaliplatin twice a week, or the corresponding solvent comprised the intraperitoneal injection. (**B**,**C**) There was a significant decrease in weight (**B**) and size (**C**) of xenografts treated with Orlistat or oxaliplatin. Compared to the oxaliplatin-resistance cells treated with oxaliplatin alone, those treated with combination of Orlistat and oxaliplatin had smaller sizes and lighter weights. (**D**) The mouse weight in each group did not have significant differences. **, *p* ≤ 0.01, *** *p* ≤ 0.001 and **** *p* ≤ 0.0001.

To verify that Orlistat can reverse oxaliplatin resistance in vivo, we performed the subcutaneous implantation of HCT116-LOHP cells in athymic nude mice. At one week postimplantation, mice were intraperitoneally injected with saline control, oxaliplatin alone, Orlistat alone, or a sequential combination of oxaliplatin and Orlistat. As expected, a measurement of tumor size and weight in mice indicated that Orlistat combined with oxaliplatin significantly reduced tumor growth (Figure 6A–C). At the end of the experiment, mean sizes of 2.04, 1.51, and 1.34 cm^3^ were reached in control, Orlistat-treated, and oxaliplatin-treated mice, respectively. Furthermore, the mean size of xenografts treated with Orlistat and oxaliplatin was 1.04 cm^3^ (*p* = 0.0065). Although oxaliplatin inhibited tumor growth, the addition of Orlistat more significantly impaired the tumorigenic ability of colorectal cancer cells. Studies have reported reduced body weight in mice treated with the fatty acid synthase inhibitors cerulenin and C75 [11]. However, in our studies, Orlistat alone did not cause weight loss, but the combination of Orlistat and oxaliplatin resulted in modest but insignificant weight loss (Figure 6D).

## 4. Discussion

Chemotherapy is an essential part of the comprehensive treatment of CRC, especially for advanced-stage patients. As a third-generation platinum drug, oxaliplatin is the cornerstone of colorectal cancer chemotherapy regimens. The derived chemotherapy regimens have helped colorectal cancer patients delay recurrence and prolong survival. However, intrinsic or acquired resistance is multifactorial and leads to treatment failure.

Many studies have proven that changes in metabolic pathways, such as glucose, fatty acids, and amino acids, are landmark events in the development of tumors [3,12]. In addition to the Warburg effect, alterations in fatty acid metabolism are related to oxaliplatin metabolism and may be a potential therapeutic target in tumor chemotherapy resistance [11]. FASN is the key enzyme in de novo lipogenesis and can be regulated by multiple transcriptional regulatory factors. A previous study revealed that regulating lipid metabolism in CRC motivates tumorigenesis, progression, and further invasion [23]. In addition, a previous study demonstrated that fatty-acid-associated genes could predict prognosis, sensitivity to 5-FU, and immunotherapy in CRC [8]. The analysis of the GEO dataset in our study also implied that FASN and closely related gene signatures were correlated with the response to oxaliplatin-based chemotherapy and a poor prognosis. Additionally, FASN was closely related to oxaliplatin resistance in CRC cell lines.

Furthermore, we hypothesized that the pharmacological inhibition of de novo lipid synthesis in colorectal cancer could overcome oxaliplatin resistance. When we used a general inhibitor of fatty acid metabolism, such as Orlistat, oxaliplatin-resistant cell lines could overcome resistance against oxaliplatin. Additionally, this synergistic effect was also evaluated with other inhibitors, such as C75. Orlistat and C75 are both FASN inhibitors. Orlistat inhibits the thioesterase function of the enzyme [19], while C75 is a synthetic cell-permeable α-methylene-γ-butyrolactone compound and interferes with the binding of malonyl-CoA to the β-ketoacyl synthase domain of FASN [24]. Therefore, the results demonstrated that the de novo synthesis of lipids is critical for resistance to oxaliplatin. Next, we found that exogenously supplied albumin-conjugated fatty acids could only partly rescue CRC cell lines from the antiproliferative effect of oxaliplatin. These results demonstrated that cancer cells rely mainly on endogenous fatty acid synthesis, and FASN could help CRC resist the anti-growth effects of oxaliplatin through the accumulation of fatty acids.

Furthermore, we sought to delineate the specific mechanisms by which fatty acid anabolism contributes to the resistance of colorectal cancer cells to the antiproliferative effects of oxaliplatin. Orlistat, an FDA-approved FASN inhibitor, also results in G1/S phase arrest and the induction of apoptosis [25]. Our study also revealed that in colorectal cancer, Orlistat caused cell cycle arrest, which triggered apoptosis. Next, RNA-seq analysis revealed that a combination of Orlistat and oxaliplatin increased apoptosis through PI3K/AKT pathways. Zhang et al. implied that Orlistat could restrain AKT/c-Met-induced hepatocarcinogenesis in vivo [26]. In our study, we also demonstrated that treatment with Orlistat led to the suppression of PI3K/AKT. Additionally, other studies reported a dual-directional connection between FASN and AKT/mTORC1 in colorectal, HCC, and ovarian carcinoma in vitro [27]. These results supported the idea that there may be a positive feedback loop strengthening AKT signaling and lipogenesis. Additionally, PI3K-Akt signaling inhibited colorectal cancer cells’ apoptosis by maintaining the stability of the cell cycle [28]. As both FASN and PI3K/AKT promote oxaliplatin resistance, the application of Orlistat can overcome oxaliplatin resistance in human CRC.

Additionally, the results of RNA-seq analysis showed that after receiving Orlistat treatment, the MAPK pathway in colorectal cancer cells was also inhibited, and the inhibition was mainly reflected in the decrease in phosphorylation levels. This result was further validated by Western blotting. A previous study demonstrated that lipid biosynthesis promoted MAPK phosphorylation [29]. ROS can reduce the release of EGFR ligands and thereby inhibit downstream pathways, including MAPK [30,31]. Our data showed that upon Orlistat treatment, ROS levels continued to increase, and the levels of phosphorylated MAPK were inhibited. Therefore, Orlistat treatment may enhance ROS levels, which blocks MAPK/ERK signaling.

Although our study highlighted that FASN enhanced oxaliplatin resistance in colorectal cancer cells, it still had some limitations. First, as both the composition of fatty acid and tumorigenesis of colorectal cancer are closely related to diet patterns, further investigation of the relationship between dietary patterns and FASN, as well as the response of chemotherapy, are warranted. Second, CRC tissue samples with the clinical data of different responses to oxaliplatin treatment would provide more evidence and deserve further study in the future.

## 5. Conclusions

In conclusion, our study depicts the relationship between FASN expression and colorectal cancer prognosis and oxaliplatin resistance. We demonstrated that de novo lipogenesis might cause oxaliplatin resistance in CRC and that inhibiting FASN and lipid synthesis could resensitize oxaliplatin-resistant cell lines by regulating the MAPK/ERK and PI3K/AKT pathways. Hence, our study produces novel insights into the mechanisms of oxaliplatin resistance and provides potential therapeutic schemes for reducing resistance to chemotherapy when managing colorectal cancer.

## Data Availability

Upon reasonable request, data and materials supporting these findings in the present study will be available from the corresponding author.

## Supplementary Materials

The following supporting information can be downloaded at: https://www.mdpi.com/article/10.3390/cancers15030562/s1, Figure S1: FASN was involved in the proliferation and invasiveness of colorectal cancer cell lines; Figure S2: Orlistat also inhibited migration and proliferation of wild-type CRC cell lines; Figure S3: Palmitate could partly rescue anti-proliferation and anti-migration effects of oxaliplatin; Figure S4: C75 inhibited lipid biosynthesis, which could overcome oxaliplatin resistance in CRC cells; Figure S5: Uncropped whole blots showing all the bands with all molecular weight markers on the Western blot; Table S1: Primer sequences for qRT-PCR; Table S2: Relative intensity ratio of each band on western blot for Figure 5C,D; Table S3: Relative intensity ratio of each band on western blot for Figure 5E,F; Table S4: Relative intensity ratio of each band on western blot for Figure S1A; Table S5: Relative intensity ratio of each band on western blot for Figure S1D.
