# Correction: Han et al. Enhanced De Novo Lipid Synthesis Mediated by FASN Induces Chemoresistance in Colorectal Cancer. *Cancers* 2023, *15*, 562

**DOI:** 10.3390/cancers17243941

**Published:** 2025-12-10

**Authors:** Lingyu Han, Weixing Dai, Wenqin Luo, Li Ye, Hongsheng Fang, Shaobo Mo, Qingguo Li, Ye Xu, Renjie Wang, Guoxiang Cai

**Affiliations:** 1 Department of Colorectal Surgery, Fudan University Shanghai Cancer Center, Shanghai 200032, China; 2 Department of Oncology, Shanghai Medical College, Fudan University, Shanghai 200032, China

## Error in Figure

In the original publication [1], there were mistakes in Figures 2, S1, S2 and S4 as published. Unintentional errors occurred during the assembly of representative images from the original figures. The corrected Figure 2, Figure S1, Figure S2 and Figure S4 appears below. The authors state that the scientific conclusions are unaffected. This correction was approved by the Academic Editor. The original publication has also been updated.

**Figure 2:**
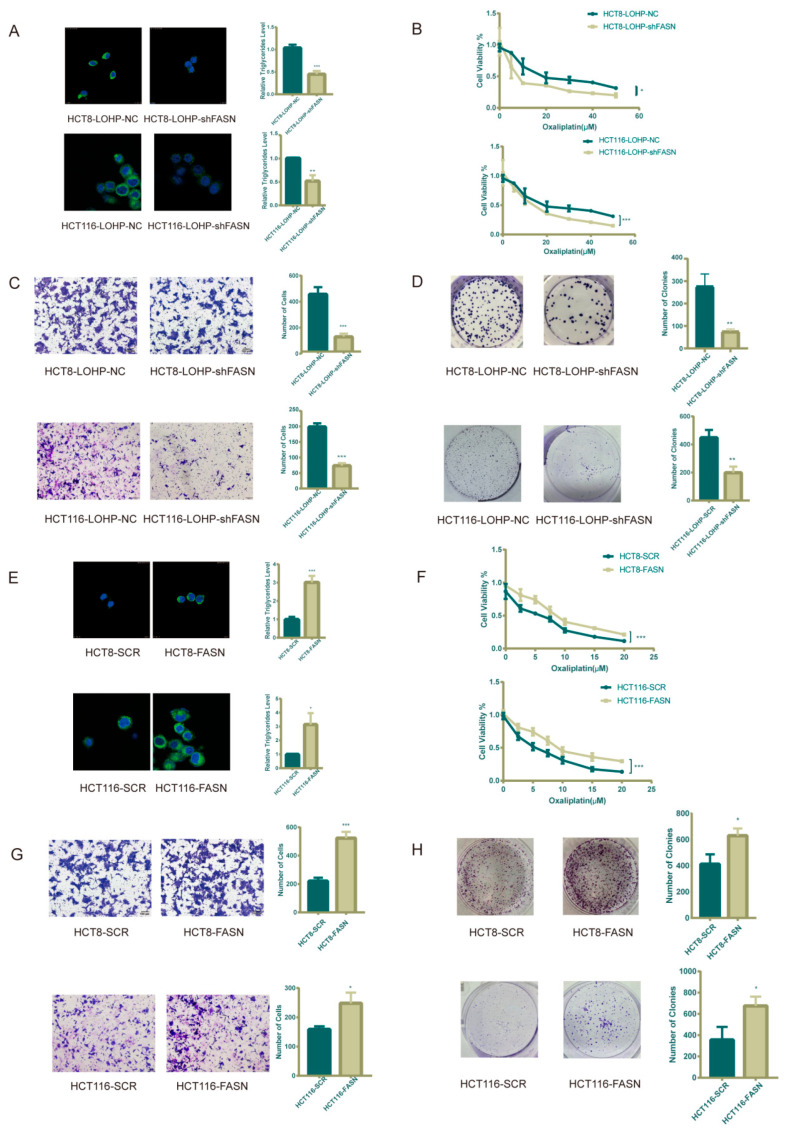
De novo lipid biosynthesis makes CRC cell lines more resistant to oxaliplatin. (**A**) Intracellular neutral lipid droplets and triglycerides were decreased in oxaliplatin-resistant cells after FASN knockdown. The storage of lipids is shown as bodipy 493/503 staining of neutral lipid droplets. (**B**) Upon treatment with same dose of oxaliplatin, there was a significant fold decrease in proliferation in the FASN knockdown CRC cells. (**C**) Transwell assay indicated that FASN knockdown inhibited migration of oxaliplatin-resistant CRC cells previously treated with 20 μM oxaliplatin for 72 h. (**D**) FASN knockdown and relative wild-type oxaliplatin-resistant CRC cells were previously treated with 20 μM oxaliplatin for 72 h. Clonogenic assay was performed as 500 cells seeded in 6-well plates for 7 days and counting. (**E**,**F**) Intracellular neutral lipid droplets, triglycerides, and IC50 of oxaliplatin increased after FASN overexpression. (**G**) Transwell assay indicated that FASN overexpression improved migration of CRC cells previously treated with 5 μM oxaliplatin for 72 h. (**H**) There was a distinct increase in colony formation in FASN-overexpression CRC cells. * *p* ≤ 0.05, ** *p* ≤ 0.01, and *** *p* ≤ 0.001.

**Supplementary Figure S1:**
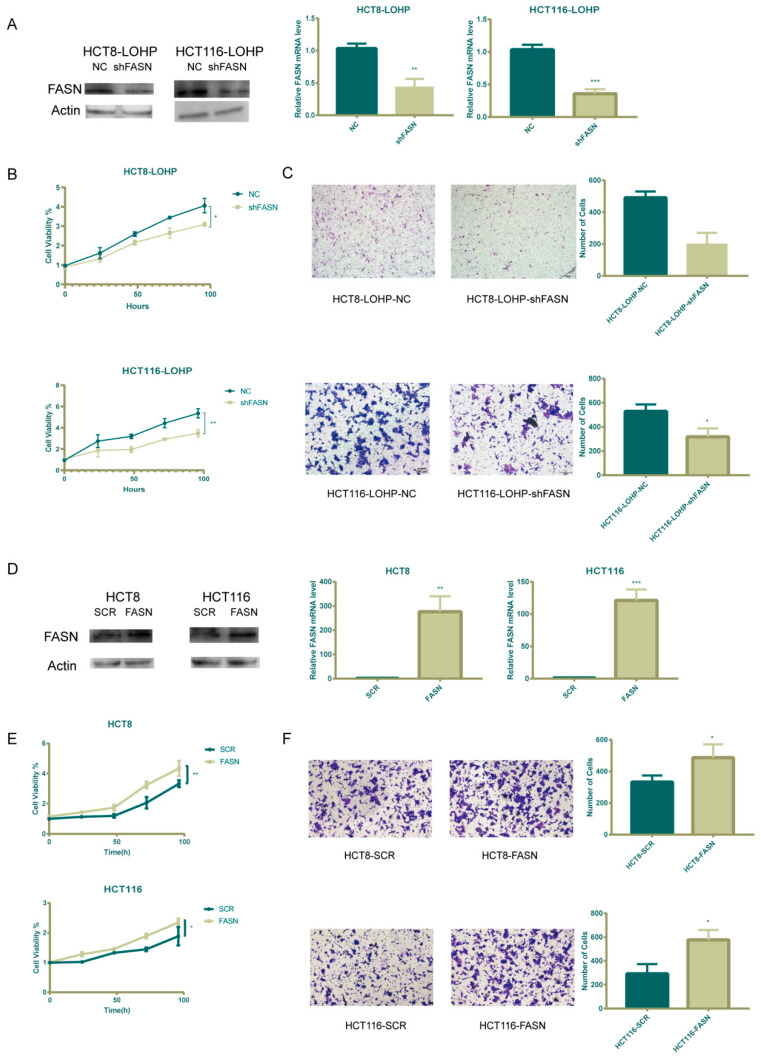
FASN was involved in the proliferation and invasiveness of colorectal cancer cell lines. A: shRNA lentiviral construct was utilized to knock down FASN in HCT116-LOHP and HCT8-LOHP cell lines, which was validated by RT-PCR and Western blotting. B: Proliferation was significantly decreased after FASN knock down. Cell viability was assessed using MTT assay. C: The migration of shFASN cells was analyzed by transwell assays. D: FASN overexpression lentivirus was transfected into HCT8 and HCT116 cell lines, which was validated by RT-PCR and Western blot. E: There was a significant increase in proliferation after FASN overexpression. F: Transwell assays indicated a robust increase in cell migration when FASN was overexpressed. *, P≤0.05; **, P≤0.01; and ***, P≤0.001.

**Supplementary Figure S2:**
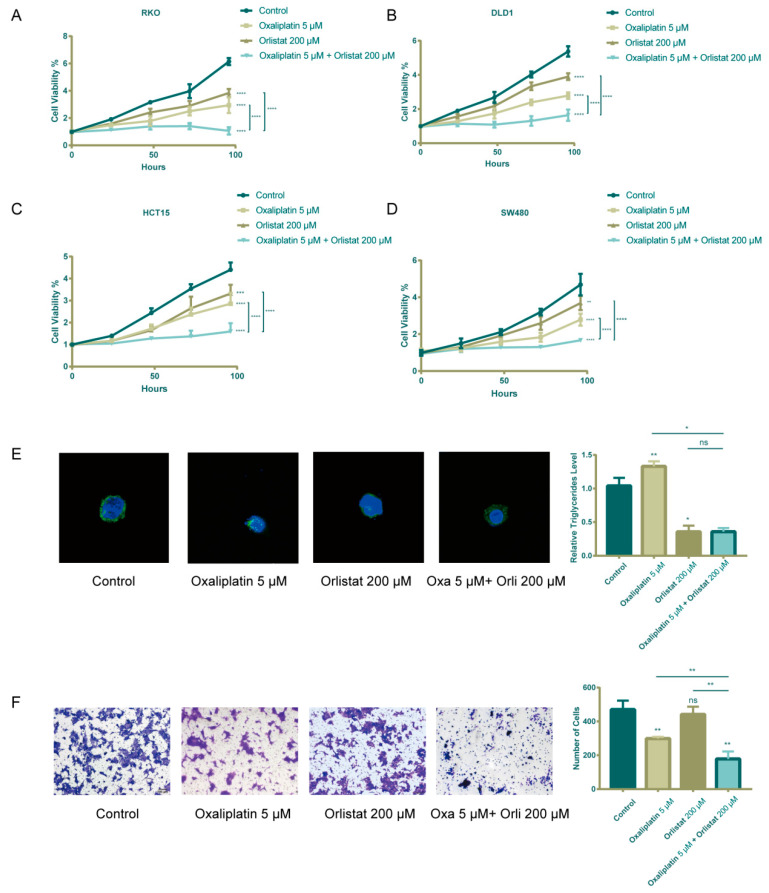
Orlistat also inhibited migration and proliferation of wild-type CRC cell lines. A–D: RKO, DLD1, HCT15, and SW480 were treated with different treatment schemes: control, 5 μM oxaliplatin, 200 μM orlistat, and combination simultaneously. Orlistat promoted oxaliplatin in cell proliferation. E: Intracellular neutral lipid droplets and triglycerides of RKO cell lines decreased after orlistat treatment. F: Transwell assay demonstrated that orlistat could inhibit RKO migration, while the inhibitory effect of orlistat alone was insignificant. Ns: no significance, *, P≤0.05; **, P≤0.01; ***, P≤0.001 and ****, p ≤ 0.0001.

**Supplementary Figure S4:**
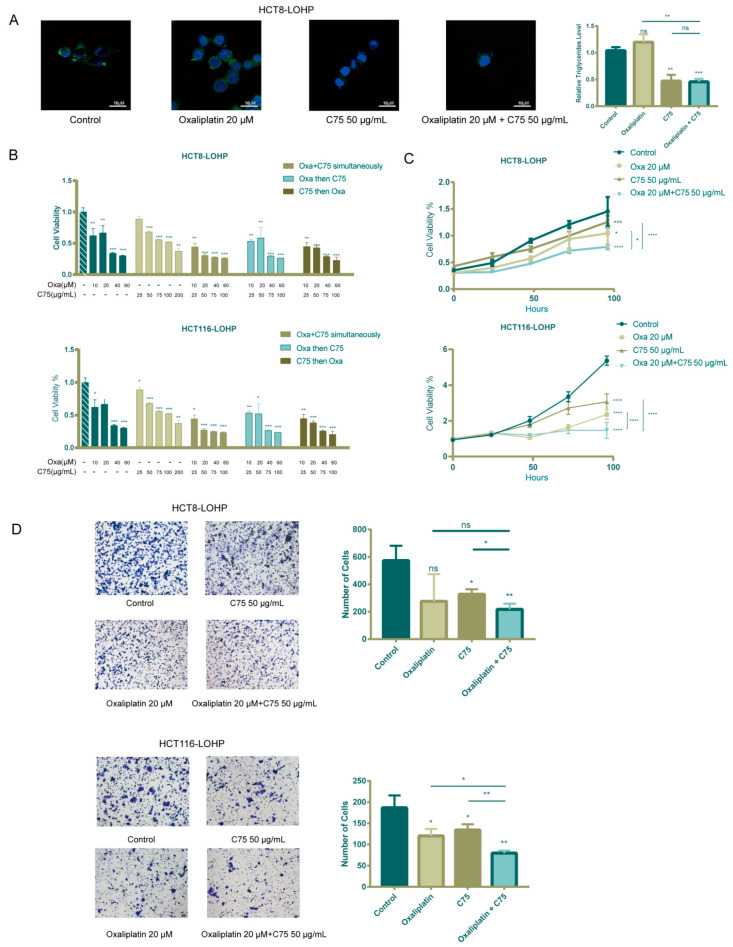
C75 inhibited lipid biosynthesis, which could overcome oxaliplatin resistance in CRC cells. A: There was a significant decrease in intracellular neutral lipid droplets and triglycerides after treatment with C75 to HCT8-LOHP for 72 hours. B and C: Both HCT8-LOHP and HCT116-LOHP were treated with different schemes for 48 hours, then 1000 cells were planted in 96-well plates. After 96 hours, proliferation of those treated with combination of C75 and oxaliplatin was robustly inhibited. D: Transwell assay indicated that C75 could also promote anti-migration effect of oxaliplatin. Ns: no significance, *, P≤0.05; **, P≤0.01; ***, P≤0.001 and ****, p ≤ 0.0001.
