# Supplementary material for: Enhanced De Novo Lipid Synthesis Mediated by FASN Induces Chemoresistance in Colorectal Cancer
Source: Cancers (Basel). 2023 Jan 17;15(3):562. doi: 10.3390/cancers15030562 (PMC9913810; doi:10.3390/cancers15030562)
Supplement: Supplementary file 1 [file cancers-15-00562-s001.zip › cancers-4036748-supplementary.pdf]

Supplementary Figure legends:

Supplementary Figure S1. FASN was involved in the proliferation and invasiveness of colorectal cancer cell lines. A: shRNA lentiviral construct was utilized to knock down FASN in HCT116-LOHP and HCT8-LOHP cell lines, which was validated by RT-PCR and Western blotting. B: Proliferation was significantly decreased after FASN knock down. Cell viability was assessed using MTT assay. C: The migration of shFASN cells was analyzed by transwell assays. D: FASN overexpression lentivirus was transfected into HCT8 and HCT116 cell lines, which was validated by RT-PCR and Western blot. E: There was a significant increase in proliferation after FASN overexpression. F: Transwell assays indicated a robust increase in cell migration when FASN was overexpressed. *, P⩽0.05; **, P⩽ 0.01; and***, P⩽0.001.

Supplementary Figure S2. Orlistat also inhibited migration and proliferation of wild-type CRC cell lines. A–D: RKO, DLD1, HCT15, and SW480 were treated with different treatment schemes: control, 5 μM oxaliplatin, 200 μM orlistat, and combination simultaneously. Orlistat promoted oxaliplatin in cell proliferation. E: Intracellular neutral lipid droplets and triglycerides of RKO cell lines decreased after orlistat treatment. F: Transwell assay demonstrated that orlistat could inhibit RKO migration, while the inhibitory effect of orlistat alone was insignificant. Ns: no significance, *, P⩽ 0.05; **, P⩽0.01; ***, P⩽0.001 and ****, p ⩽ 0.0001.

Supplementary Figure S3. Palmitate could partly rescue anti-proliferation and anti-migration effects of oxaliplatin. A: After being treated for 72 hours, CCK8 assays indicated that cell viability of both HCT8-LOHP and HCT116-LOHP cell lines increased with additional treatment with palmitate. B and C: With treatment with 20 μM oxaliplatin, palmitate could partly rescue anti-proliferation and anti-migration of orlistat. Ns: no significance, *, P⩽0.05; **, P⩽0.01; ***, P⩽0.001 and ****, p ⩽ 0.0001.

Supplementary Figure S4. C75 inhibited lipid biosynthesis, which could overcome oxaliplatin resistance in CRC cells. A: There was a significant decrease in intracellular neutral lipid droplets and triglycerides after treatment with C75 to HCT8-LOHP for 72 hours. B and C: Both HCT8-LOHP and HCT116-LOHP were treated with different schemes for 48 hours, then 1000 cells were planted in 96-well plates. After 96 hours, proliferation of those treated with combination of C75 and oxaliplatin was robustly inhibited. D: Transwell assay indicated that C75 could also promote anti-migration effect of oxaliplatin. Ns: no significance, *, P⩽0.05; **, P⩽0.01; ***, P⩽0.001 and ****, p ⩽ 0.0001 .

Supplementary Figure S5. Uncropped whole blots showing all the bands with all molecular weight markers on the Western blot. A: Western blot indicates that phosphorylation of MAPK/ERK and PI3K/AKT pathways was inhibited by Orlistat. B: Western blot indicates the changes in FASN expression after intervention.

Supplementary Figure S1.

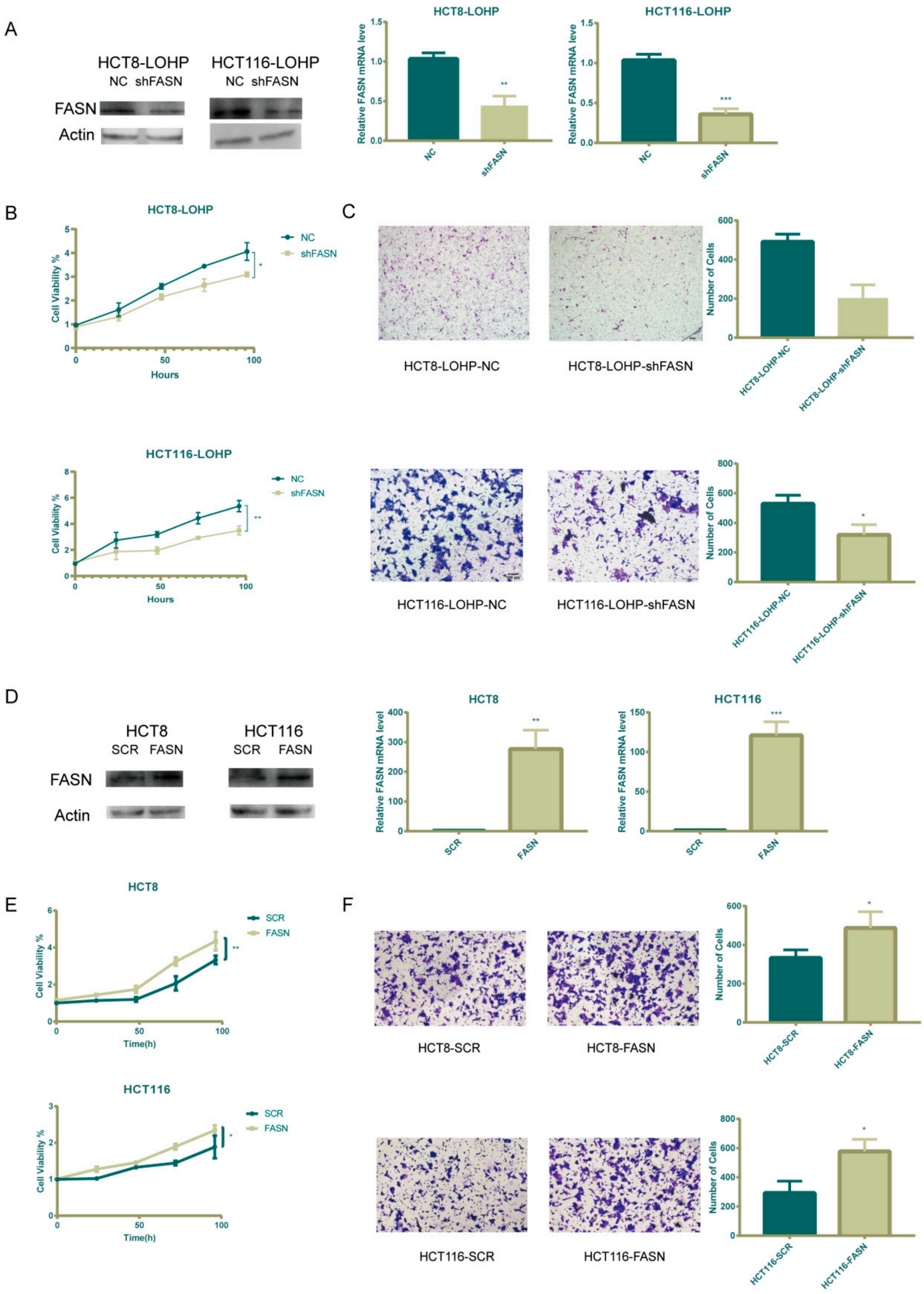

Supplementary Figure S2.

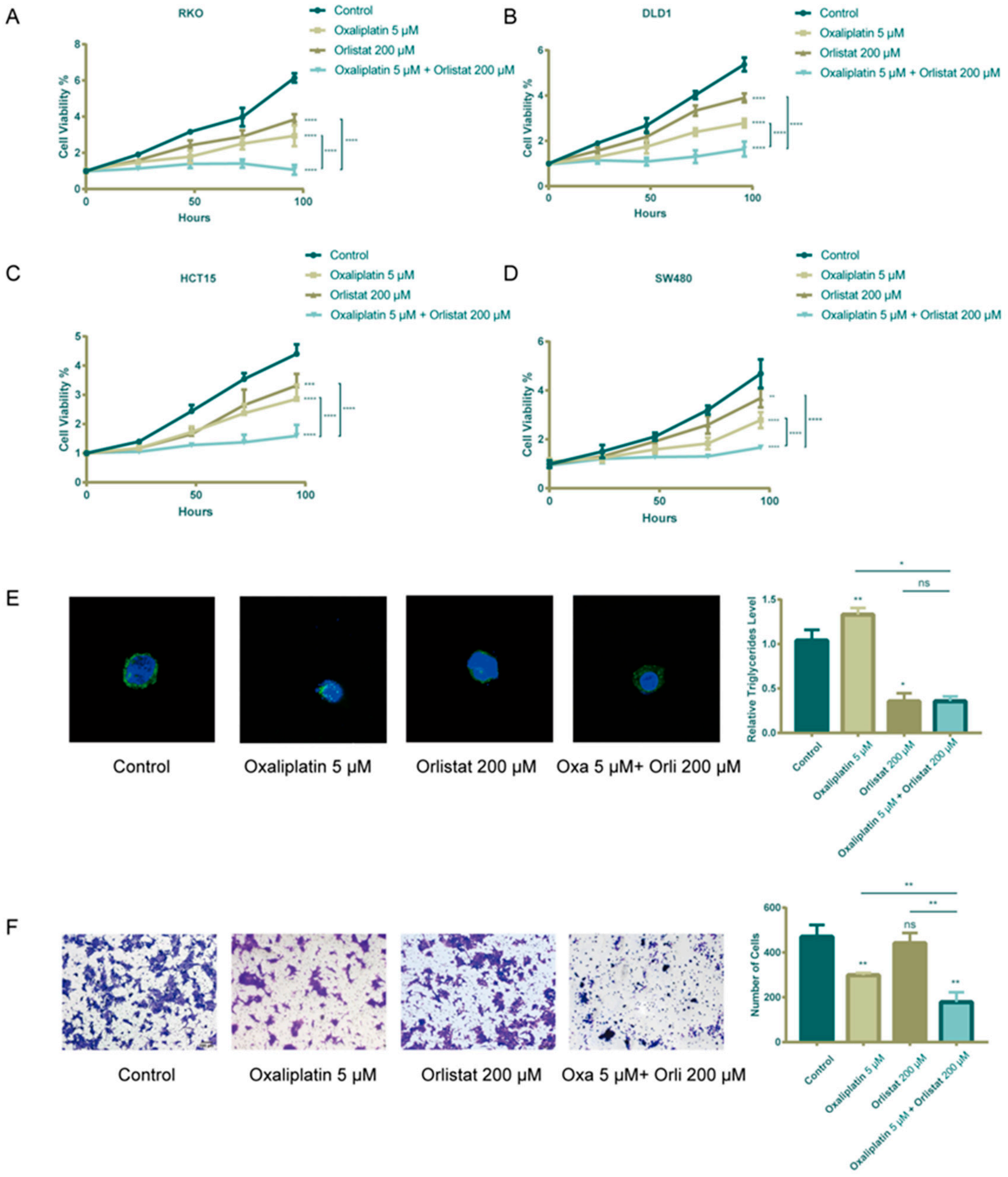

Supplementary Figure S3.

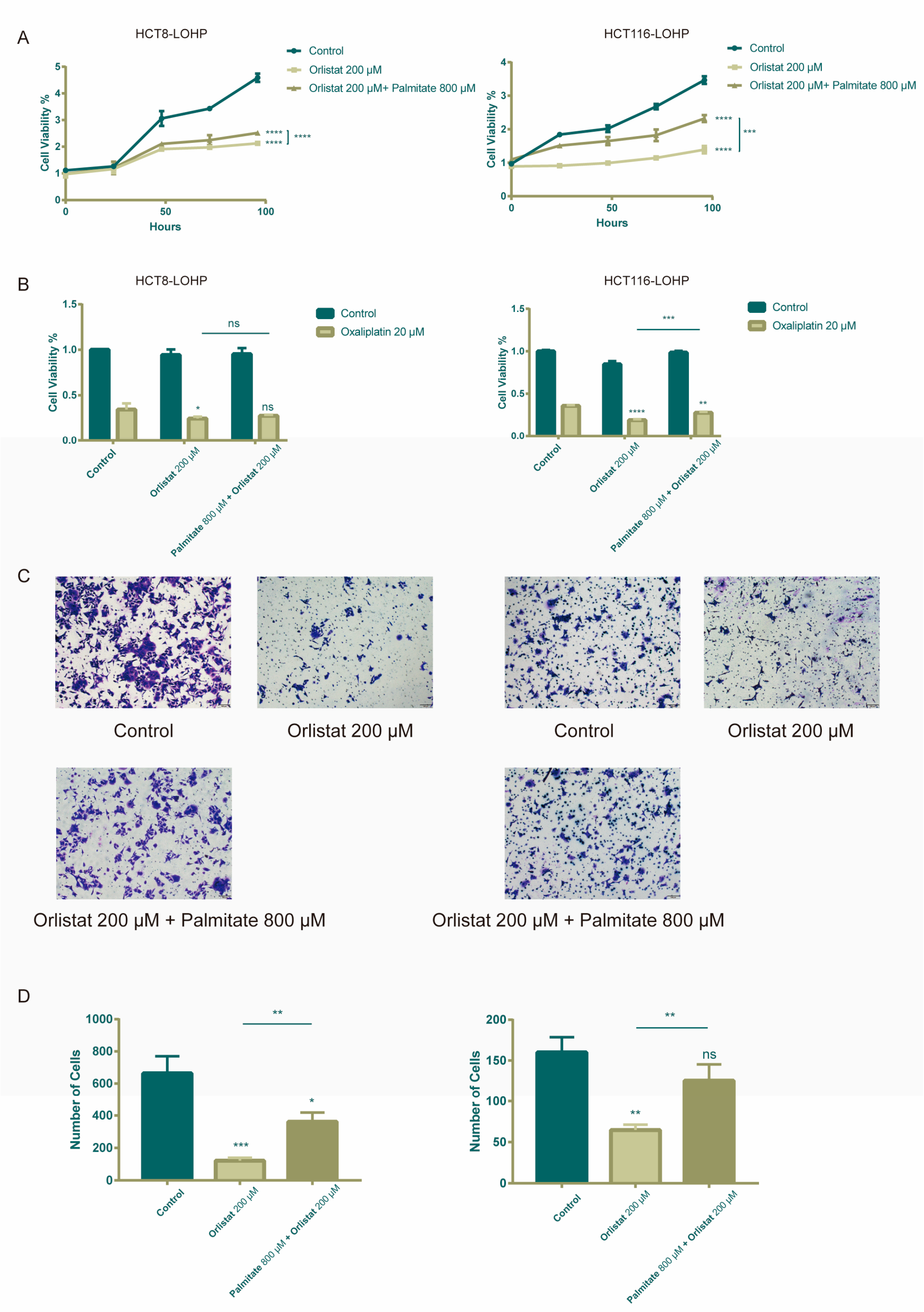

Supplementary Figure S4.

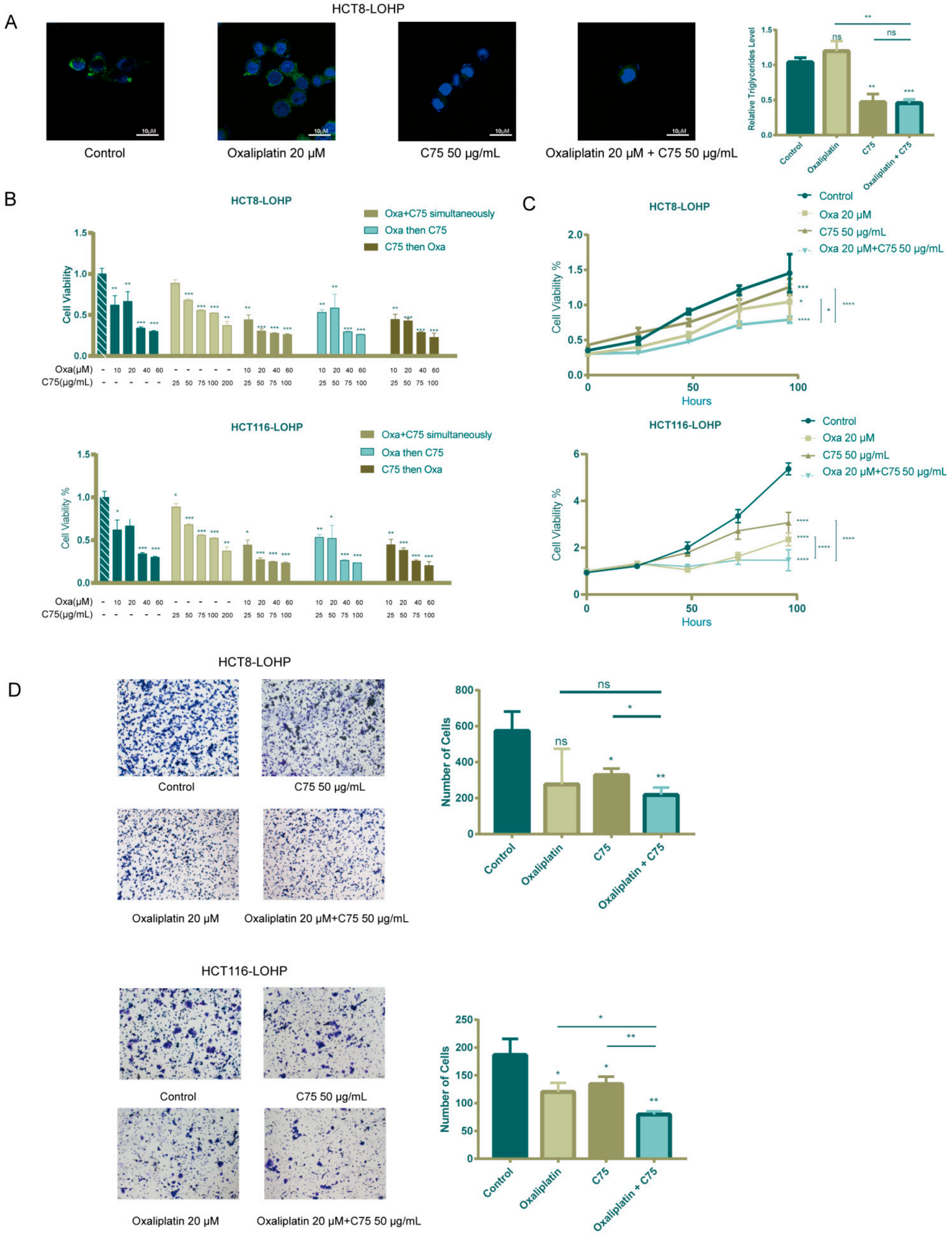

Supplementary Figure S5.

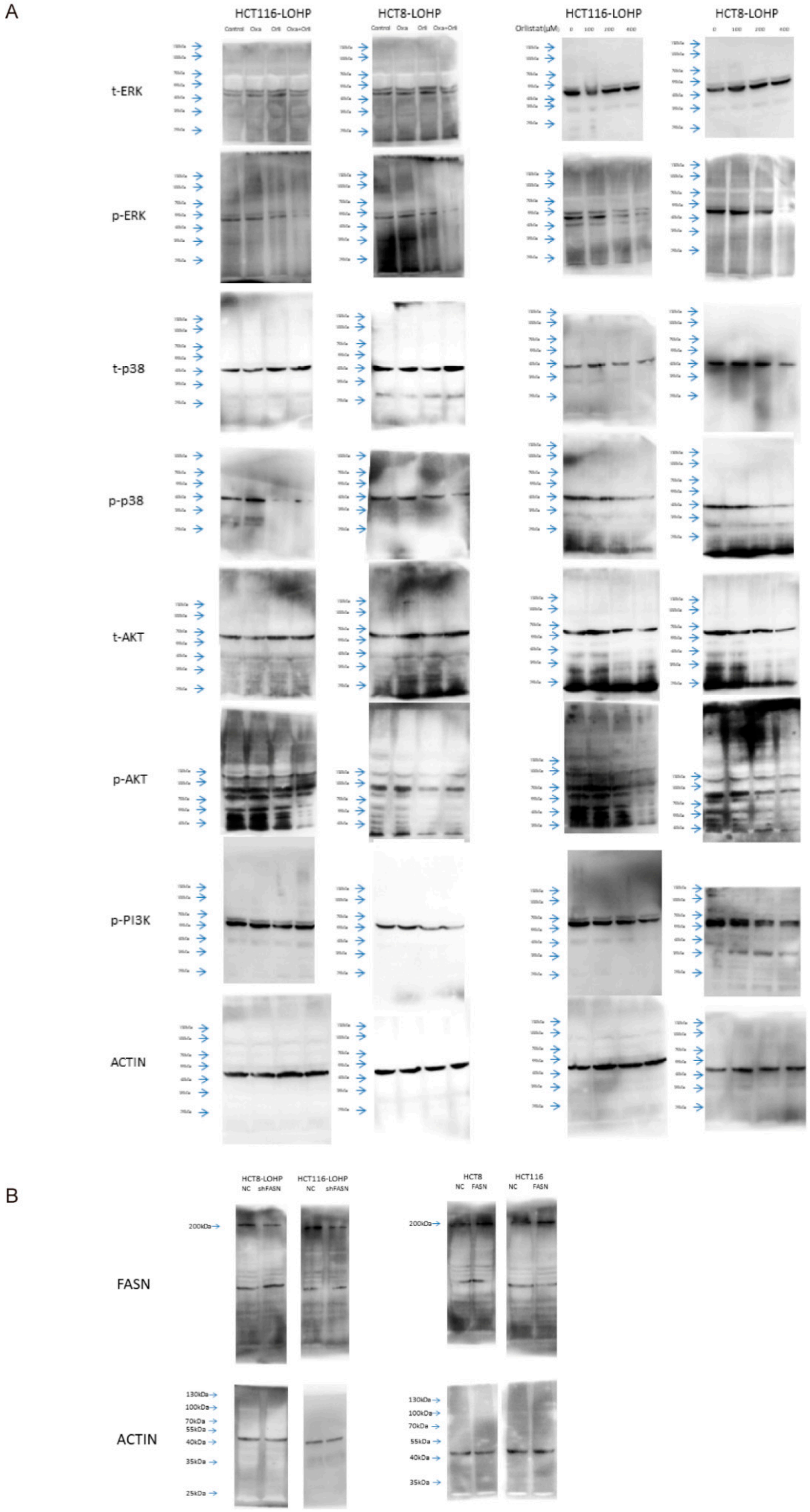

Supplemental Table S1. Primer sequences for qRT-PCR.

Gene	Species	NCBI Locus ID	Forward primer sequence (5'→3')	Reverse primer sequence (5'→3')
ACTIN	Human	NM_001101.3	CACTCTTCCAGCCTTCCTTC	GTACAGGTCTTTGCGGATGT
FASN	Human	NM_004104.5	GCAAGCTGAAGGACCTGTCT	AATCTGGGTTGATGCCTCCG

Supplemental Table S2. Relative intensity ratio of each band on western blot for Figure 5(C) and Figure 5(D).

	HCT116-LOHP				HCT8-LOHP			
	Control	Oxaliplatin	Orlistat	Oxa+Orli	Control	Oxaliplatin	Orlistat	Oxa+Orli
	1.000	1.054	0.487	0.375	1.000	1.054	0.487	0.375
p-ERK/t-ERK	1.082	0.928	0.395	0.398	0.928	0.928	0.599	0.498
	0.998	1.193	0.490	0.413	1.129	1.284	0.673	0.348
	1.098	0.982	0.294	0.498	1.083	1.202	0.736	0.674
p-p38/t-p38	0.937	1.294	0.499	0.294	0.984	0.873	0.835	0.689
	1.124	1.325	0.598	0.390	0.993	1.123	0.763	0.728
	1.000	1.219	0.706	0.475	1.000	0.810	0.620	0.725
p-AKT/t-AKT	1.103	1.240	0.872	0.699	0.982	0.928	0.893	0.649
	0.927	1.240	0.793	0.549	1.023	1.240	0.734	0.710

Supplemental Table S3. Relative intensity ratio of each band on western blot for Figure 5(E) and Figure 5(F).

	HCT116-LOHP				HCT8-LOHP			
Orlistat(μM)	0	100	200	400	0	100	200	400
p-ERK/t-ERK	1.000	0.803	0.409	0.283	1.001	1.036	0.665	0.475
	0.992	0.913	0.348	0.230	0.982	0.938	0.788	0.378
	1.092	0.784	0.361	0.294	1.103	0.835	0.587	0.460
p-p38/t-p38	1.019	0.893	0.832	0.732	1.001	0.887	0.883	0.535
	0.989	0.922	0.883	0.849	0.998	0.932	0.759	0.639
	1.123	0.979	0.921	0.839	1.013	0.872	0.929	0.573
	1.000	0.643	0.816	0.494	1.000	0.843	0.816	0.634
p-AKT/t-AKT	0.983	0.634	0.758	0.598	1.023	0.932	0.899	0.694
	1.123	0.653	0.605	0.524	0.989	0.784	0.739	0.589

Supplemental Table S4. Relative intensity ratio of each band on western blot for Figure S1(A).

HCT8-LOHP		HCT116-LOHP	
NC	shFASN	NC	shFASN
1.0273	0.7842	1.0128	0.7834
0.9982	0.8734	0.9823	0.6894
0.9823	0.6895	1.0053	0.5896

Supplemental Table S5. Relative intensity ratio of each band on western blot for Figure S1(D).

HCT8-LOHP		HCT116-LOHP	
SCR	FASN	SCR	FASN
1.021	4.398	1.0293	3.3984
0.983	4.092	0.9823	3.982
0.998	3.2982	0.9938	4.984
